# STAT3 balances myocyte hypertrophy vis-à-vis autophagy in response to Angiotensin II by modulating the AMPKα/mTOR axis

**DOI:** 10.1371/journal.pone.0179835

**Published:** 2017-07-07

**Authors:** Lei Chen, Lin Zhao, Anweshan Samanta, Seyed Morteza Mahmoudi, Tanner Buehler, Amy Cantilena, Robert J. Vincent, Magdy Girgis, Joshua Breeden, Samuel Asante, Yu-Ting Xuan, Buddhadeb Dawn

**Affiliations:** Division of Cardiovascular Diseases, Cardiovascular Research Institute, University of Kansas Medical Center, Kansas City, Kansas, United States of America; Virginia Commonwealth University Medical Center, UNITED STATES

## Abstract

Signal transducers and activators of transcription 3 (STAT3) is known to participate in various cardiovascular signal transduction pathways, including those responsible for cardiac hypertrophy and cytoprotection. However, the role of STAT3 signaling in cardiomyocyte autophagy remains unclear. We tested the hypothesis that Angiotensin II (Ang II)-induced cardiomyocyte hypertrophy is effected, at least in part, through STAT3-mediated inhibition of cellular autophagy. In H9c2 cells, Ang II treatment resulted in STAT3 activation and cellular hypertrophy in a dose-dependent manner. Ang II enhanced autophagy, albeit without impacting AMPKα/mTOR signaling or cellular ADP/ATP ratio. Pharmacologic inhibition of STAT3 with WP1066 suppressed Ang II-induced myocyte hypertrophy and mRNA expression of hypertrophy-related genes ANP and β-MHC. These molecular events were recapitulated in cells with STAT3 knockdown. Genetic or pharmacologic inhibition of STAT3 significantly increased myocyte ADP/ATP ratio and enhanced autophagy through AMPKα/mTOR signaling. Pharmacologic activation and inhibition of AMPKα attenuated and exaggerated, respectively, the effects of Ang II on ANP and β-MHC gene expression, while concomitant inhibition of STAT3 accentuated the inhibition of hypertrophy. Together, these data indicate that novel nongenomic effects of STAT3 influence myocyte energy status and modulate AMPKα/mTOR signaling and autophagy to balance the transcriptional hypertrophic response to Ang II stimulation. These findings may have significant relevance for various cardiovascular pathological processes mediated by Ang II signaling.

## Introduction

Pathological hypertrophy of cardiac muscle is one of the most common causes of heart failure in the United States [1]. The hallmark of pathological cardiac hypertrophy is enlargement of myocytes leading to increased ventricular mass with concomitant changes in the extracellular matrix. This is often secondary to increased pressure or volume overload, and is characterized at the molecular level by enhanced protein synthesis and re-expression of fetal genes such as atrial natriuretic peptide (ANP), brain natriuretic peptide (BNP) and β-myosin heavy chain (MHC) [2,3]. Understanding the molecular basis of pathological myocyte hypertrophy is a crucial prerequisite toward formulating therapeutic strategies to prevent cardiac hypertrophy and consequent heart failure.

Angiotensin II (Ang II), an endogenous peptide, is a key component of the renin-angiotensin system (RAS) that plays important roles in fluid and electrolyte homeostasis, regulation of blood pressure and pathogenesis of heart failure. Inhibition of RAS has proven highly efficacious not only for blood pressure control, but also for the attenuation of postinfarct ventricular remodeling. Although complex signaling initiated by Ang II plays an important role in pathological cardiac hypertrophy [4], the molecular details of downstream events that ultimately result in myocyte enlargement remain incompletely understood. Since hypertrophy involves a net gain in myocyte volume, the balance between increased myocyte protein synthesis with increased protein misfolding and abnormal protein degradation seems to play a key role in this process. Because of its direct impact on removal of misfolded proteins, myocyte catabolic processes, and maintenance of cellular homeostasis, autophagy has been implicated in regression of pathological myocyte hypertrophy [5]. In the setting of cardiac pressure overload-induced hypertrophy [6] and cardiac proteinopathy [7] enhanced autophagy has been shown to be an important protective mechanism for removal of excessive and misfolded proteins. However, the role of autophagy during Ang II-induced myocyte hypertrophy remains poorly understood.

In hypertrophic hearts, the source of energy in the form of ATP switches from fatty acid oxidation to increased glucose utilization with decrease in ATP levels. Activated by increased AMP/ATP or ADP/ATP ratio, AMPKα is an important mediator of metabolic adaptation that promotes ATP production. AMPKα activation results in inhibition of mammalian target of rapamycin (mTOR), which is important for both energy usage and inhibition of autophagy [8,9]. Although STAT3 colocalizes with complex I of the respiratory chain and is essential for the activity of enzyme complexes in the electron transport chain [10], the aggregate role of STAT3 in myocyte metabolism, autophagy, and hypertrophy remains poorly understood. Indeed, no study has examined the effects of STAT3 on the activation of AMPKαand how it relates to myocyte hypertrophy. Furthermore, the role of STAT3 in Ang II-induced myocyte autophagy has not been investigated.

We hypothesized that Ang II-induced myocyte hypertrophy results, at least in part, from STAT3-mediated suppression of myocyte autophagy. This hypothesis was tested in H9c2 cells using both pharmacologic and genetic inhibition of STAT3 as well as activation and inhibition of AMPKα/mTOR signaling axis. Our results indicate that although STAT3 activation is necessary for Ang II-induced myocyte hypertrophy, its inhibition tilts the balance toward autophagy promotion and amelioration of hypertrophy through activation of AMPKα.

## Materials and methods

### Media, reagents, antibodies

Media and reagents were acquired from the following sources: DMEM and Trypsin from Corning; FBS from Gibco; cell lysis buffer from Cell Signaling Technology; angiotensin II, WP1066, AICAR and compound C from EMD Millipore; (GFP)-fused LC3B (pEGFP-LC3) from Addgene; and ADP/ATP assay kit from Sigma. Antibodies were acquired from the following sources: rabbit anti-p-STAT3 (Tyr705), rabbit anti-p-STAT3 (Ser727), rabbit anti-STAT3, rabbit anti-p-mTOR (Ser2448), anti-mTOR, rabbit anti-p-AMPKα (T172), rabbit anti-AMPKα, rabbit anti-p-JAK2 (Tyr1007/1008) and rabbit anti-p-4E-BP1 (T37/46) from Cell Signaling; rabbit anti-LC3Bfrom Novus Biologicals; rabbit anti-β-Actin and rabbit anti-p62 from Santa Cruz Biotechnology.

### Cell culture

H9c2 cells were purchased from ATCC (ATCC^®^CRL-1446™) and maintained in DMEM with 4.5g/L glucose supplemented with 10% (v/v) FBS. Cells were expanded in cultured to 80% confluence prior to passage and harvest for experiments following standard protocols. Before experiments, cells were plated in growth media for 24 h. Cells were exposed to Ang II alone or in combination with other agents (WP1066, AICAR, Compound C), which were added to the culture media at various concentrations for 48 h.

### Generation of stable STAT3 knockdown cells

HEK293 cells were transfected with piLenti-shRNA-STAT3 vector (target 1135: AGAGGGTCTCGGAAATTTAACATTCTGGG, Applied Biological Materials, i065664) and the resulting medium was used to infect H9c2 cells for 24 h. This was followed by puromycin selection for 7 d. Single colonies were then picked and amplified on puromycin selective media.

### Cell treatment

H9c2 cells were treated with 5 μM of Ang II in culture medium for 12, 24 or 48 h. To further explore the mechanism by which STAT3 affected Ang II-induced cellular hypertrophy, cells were treated with 1 mM of AICAR (specific AMPKα activator) or 5 μM of Compound C (specific AMPKα inhibitor) and co-treated with Ang II (5 μM) and/or WP1066 (4 μM). All experiments were performed in triplicate.

### Assessment of cellular hypertrophy

H9c2 cells were fixed with 4% paraformaldehyde and stained with 1% crystal violet (Fisher Scientific). The cross-sectional surface area of at least 100 cells in each experimental group was measured by a blinded investigator in microscopic images acquired from at least 10 random fields using the Image-Pro Plus software (Media Cybernetics).

### Western immunoblotting

Western immunoblotting analysis was performed using standard sodium dodecyl sulfate-polyacrylamide gel electrophoresis techniques to determine levels of p-mTOR rabbit polyclonal antibody (#2971, dilution 1:1000), mTOR rabbit polyclonal antibody (#2972, dilution 1:1000), p-STAT3 (Tyr705)rabbit monoclonal antibody (#9145, dilution1:1000), p-STAT3 (Ser727)rabbit polyclonal antibody (#9134, dilution 1:1000), STAT3 rabbit monoclonal antibody (#12640, dilution 1:1000), p-AMPKα (T172)rabbit monoclonal antibody (#2535, dilution 1:1000), AMPKαrabbit polyclonal antibody (#2532, dilution 1:1000), p-JAK2rabbit monoclonal antibody (#3776, dilution 1:1000), p-4E-BP1 Rabbit monoclonal antibody (#2855, dilution 1:1000) (Cell Signaling Technology),LC3-I and -II rabbit polyclonal Ab (NB100-2220, dilution 1:1000) (Novus Biologicals), β-Actin goat polyclonal antibody (sc-1616, dilution 1:1000), anti-p62 rabbit polyclonal antibody (sc-25523, dilution 1:500) (Santa Cruz Biotechnology). Incubation of infrared dye-labeled secondary antibodies (LI-COR Biotechnology) was performed in darkness for 1 h at room temperature. After extensive washing, membranes were scanned and analyzed on an Odyssey™ scanner (LI-COR Biotechnology).

### Subcellular localization of EGFP-LC3 protein

H9c2 cells were transfected with plasmid encoding EGFP-LC3 (EGFP-labeled microtubule-associated protein 1A/1B-light chain 3) [11] and selected with puromycin for stable expression. After exposure to Ang II and/or WP1066 for 48 h, the EGFP-LC3 puncta were analyzed using confocal microscopy.

### Electron microscopy

H9c2 cells transfected with scramble control or STAT3 shRNA, H9c2 cells were fixed with 2.5% glutaraldehyde (in 0.1 M Sodium Cacodylate Buffer, pH 7.4), dehydrated in a graded series of ethanol, and critical point dried in EMS 850 Critical Point Drier. Samples were mounted on Al mounts and sputter-coated with gold in the EMS 150 RES sputter coater and examined using Hitachi S-2700 scanning electron microscope.

### ADP/ATP assay

The ADP/ATP ratio was measured to evaluate the energy status of cultured H9c2 cells using a commercially available kit (Sigma). Briefly, after exposure to various agents for specified times, working reagent was added to culture medium to lyze cells and release ADP and ATP. ATP levels were measured in relative light units following interaction between ATP and substrate (D-luciferin) in presence of luciferase. After 10 min, ADP is converted to ATP and the total ATP levels are measured under the same wavelength of plate reader (BioTeck, Synergy HT). The ADP/ATP ratio was calculated from these numbers following standard protocol.

### Quantitative real-time RT-PCR

Following exposure to various agents in culture, cellular total RNA was isolated using TRIzol Reagent (Invitrogen). qRT-PCR was performed following standard methods using SYBR green (Applied Biosystems). The following primer sequences were used: GAPDH, 5’-ATGGGAAGCTGGTCATCAAC-3’ (forward) and 5’-GTGGTTCACACCCATCACAA-3’ (reverse); ANP, 5’-ATACAGTGCGGTGTCCAACA-3’ (forward) and 5’-AGCCCTCAGTTTG CTTTTCA-3’ (reverse); β-MHC, 5’-GGAGAAAGAGAAGAGCGAGTTC-3’ (forward) and 5’-GGCACATCTTCTCCAGGTTAG-3’ (reverse). PCR reactions were performed using the following conditions: denaturation at 95°C (5 min) and 45 cycles for GAPDH, ANP and β-MHC consisting of 3s (95°C), 30s (60°C), and 30s (72°C). All data were normalized to GAPDH and quantitative measurements were obtained using the ΔΔ-CT method.

### Statistical analysis

Data were expressed as mean ± SEM. For comparison between 2 groups, 2-tailed Student’s *t*-test was used; for comparison among multiple groups (≥3groups) one-way ANOVA with Bonferroni’s *post hoc* test was used. A *P* value less than 0.05 was considered statistically significant. All statistical analyses were performed using the SPSS software version 22.0 (IBM, Armonk, NY).

## Results

### Ang II activated STAT3 and induced myocyte hypertrophy

**Fig 1:**
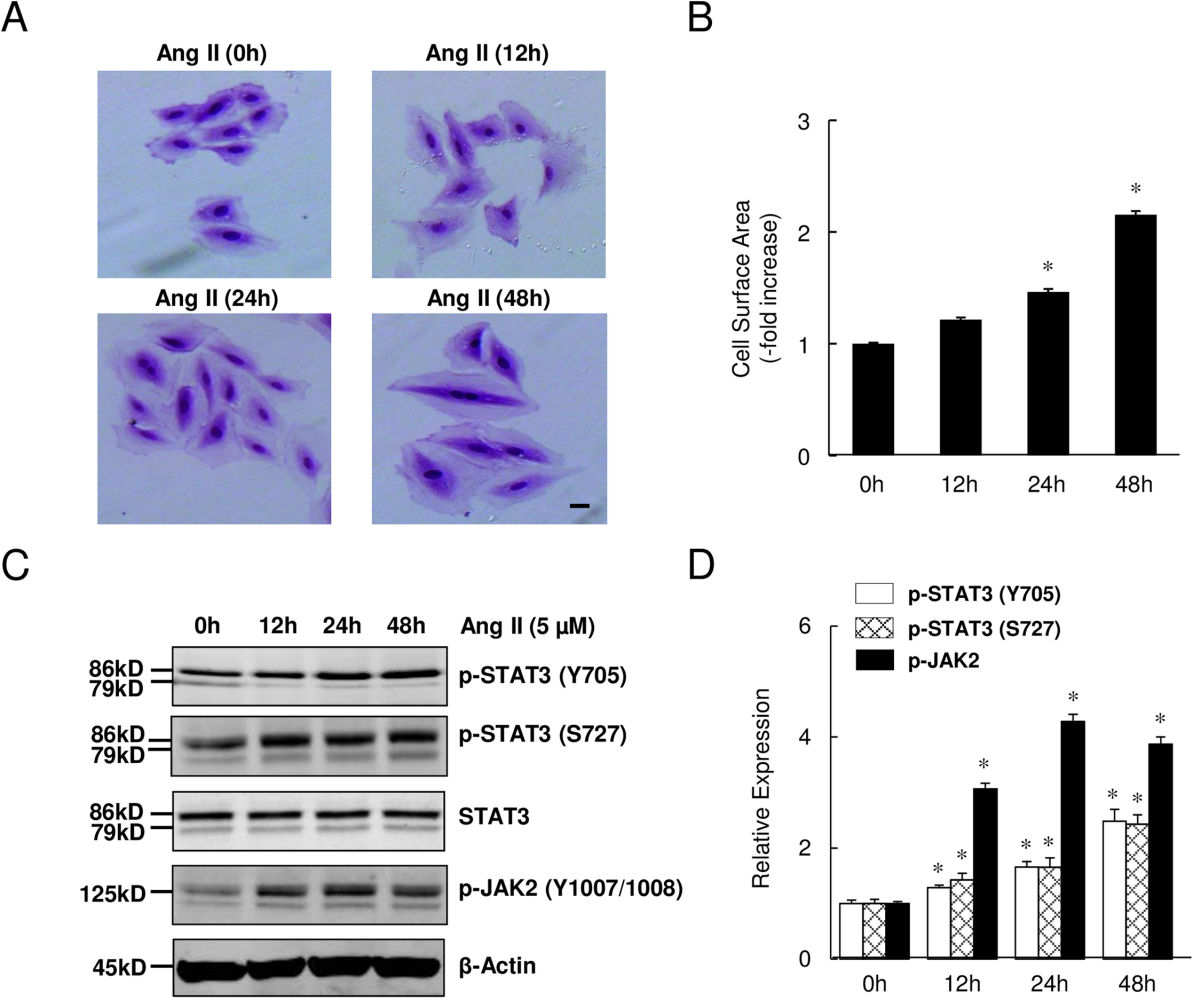
Exposure to Ang II induced myocyte hypertrophy and activated STAT3. (A) Representative photomicrographs of H9c2 cells stained with crystal violet, magnification: 20x; scale bar = 10 μm. (B) Cross-sectional cell surface area (n = 100 cells/group). (C) Time-course of Ang II-induced Tyr705 and Ser727 phosphorylation of STAT3 in H9c2 cells. (D) Densitometric data from Western immunoblotting. Levels of p-STAT3 (Y705), p-STAT3 (S727), STAT3 and p-JAK2 (Y1007/1008) were quantified and normalized relative to β-Actin. Data represent mean ± SEM (n = 4), *P<0.05 vs. baseline (0h).

As shown in Fig 1A, H9c2 cell surface area increased progressively with Ang II exposure. This increase in cell surface area was especially prominent after 48 h. The average cell surface area increased 1.22-, 1.46- and 2.15-fold after 12, 24 and 48 h of Ang II exposure, respectively, compared with untreated control cells (Fig 1B), indicating a time-dependent induction of myocyte hypertrophy by Ang II. Furthermore, Western immunoblot analysis of cellular lysates after Ang II treatment for various durations revealed increased levels of p-JAK2 as well as p-STAT3(Tyr705) and p-STAT3(Ser727) (Fig 1C), the usual phosphorylation sites for STAT3 activation. Densitometric analysis showed that p-STAT3(Tyr705), p-STAT3(Ser727), and p-JAK2 levels increased with Ang II treatment in a time-dependent manner (Fig 1D). These data indicate that Ang II treatment activates the JAK2-STAT3 axis in H9c2 cells.

### Generation of H9c2 cells with stable STAT3 suppression

**Fig 2:**
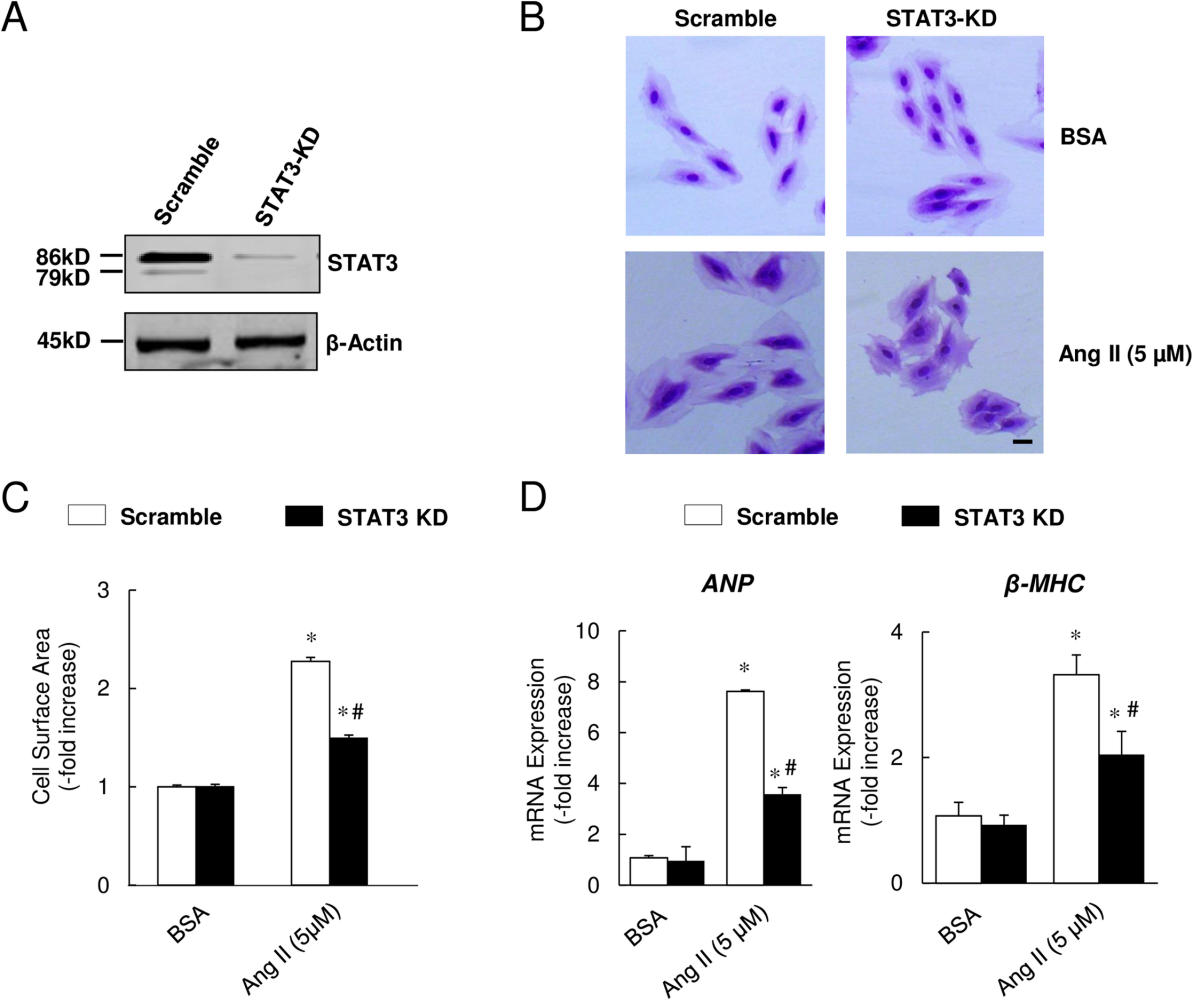
STAT3 knock-down ameliorated Ang II-induced myocyte hypertrophy. (A) Western immunoblot shows marked reduction in STAT3 expression in shRNA-treated myocytes. (B) Representative photomicrographs of H9c2 cells stained with crystal violet. Magnification 20x; scale bar = 10 μm. (C) Cross-sectional cell surface area (n = 100 cells/group). (D) qRT-PCR analysis of ANP and β-MHC mRNA expression in BSA- or Ang II-treated cells with or without STAT3 knock-down. Data represent mean ± SEM (n = 4), *P<0.05 vs. control (BSA), ^#^*P*<0.05 vs. Ang II + Scramble peptide.

In order to block endogenous STAT3 expression, STAT3-specific shRNA was used to knock down STAT3 expression. After lentiviral transfection and puromycin selection, single-cell colonies were selected and amplified. Western immunoblotting showed marked reduction in STAT3 protein expression levels in cells transfected with STAT3 shRNA compared with scramble RNA-transfected cells (Fig 2A). These cells with stable and markedly reduced STAT3 expression are referred to as STAT3-knockdown (STAT3-KD) cells.

### Inhibition of STAT3 expression attenuated Ang II-induced hypertrophy

As shown in Fig 2B, myocyte hypertrophy following Ang II treatment was significantly attenuated in STAT3-KD cells compared with cells transfected with scramble control. The quantitative analysis showed that following stimulation with Ang II, the -fold increase in average surface area of STAT3-KD cells was significantly smaller compared with the scramble control group (1.50-fold increase in STAT3-KD vs. 2.27-fold increase in scramble control group, *P*<0.05,Fig 2C), indicating that STAT3 knockdown protects against Ang II-induced myocyte hypertrophy.

Using quantitative real-time RT-PCR, we further examined the mRNA expression levels of ANP and β-MHC, molecular markers associated with cardiac hypertrophy. As shown in Fig 2D, mRNA expression levels of ANP and β-MHC increased significantly after Ang II treatment in both STAT3-KD and scramble control groups. However, STAT3 knockdown significantly blunted this increase in ANP and β-MHC levels in STAT3-KD cells compared with controls (ANP: 3.58-fold increase in STAT3-KD group vs. 7.62-fold increase in scramble group; β-MHC: 2.05-fold increase in STAT3-KD vs. 3.32-fold increase in scramble group, *P*<0.05 for both comparisons, Fig 2D). These results indicate that Ang II-induced myocyte hypertrophy involves STAT3 signaling.

### Pharmacological inhibition of STAT3 attenuated Ang II-induced hypertrophy

**Fig 3:**
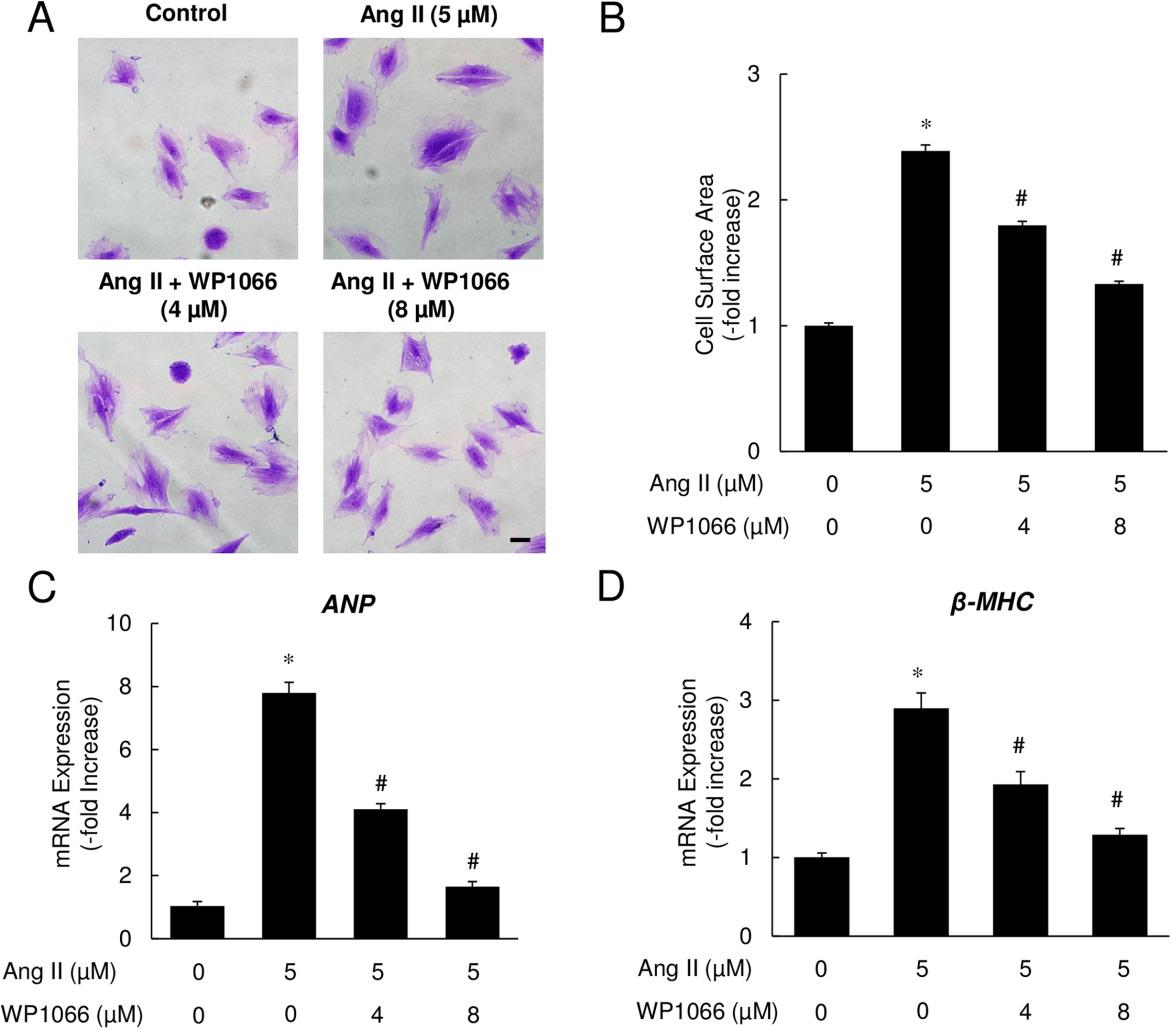
Pharmacological inhibition of STAT3 attenuated Ang II-induced myocyte hypertrophy. (A) Representative photomicrographs of H9c2 cells stained with crystal violet. Magnification 20x; scale bar = 10 μm. (B) Cross-sectional cell surface area (n = 100 cells/group). (C and D) qRT-PCR analysis of ANP and β-MHC mRNA expression in Ang II-treated cells with or without STAT3 inhibition. Data represent mean ± SEM (n = 4), *P<0.05 vs. control, ^#^*P*<0.05 vs. Ang II only.

Next, we tested whether pharmacological inhibition of STAT3 would recapitulate the above findings with genetic inhibition of STAT3 expression. As shown in Fig 3A, the addition of WP1066, a specific STAT3 inhibitor, prevented Ang II-induced hypertrophy in H9c2 cells in a dose-dependent manner. Quantitative assessment showed a 2.39-fold increase in average surface area of cells exposed to Ang II, while this increase was reduced to 1.80- and 1.33-folds with WP1066 treatment at 4 μM and 8 μM concentrations, respectively (Fig 3B).

The measurement of ANP and β-MHC mRNA levels provided further evidence indicating that STAT3 regulates Ang II-induced hypertrophy. As shown in Fig 3C and 3D, after stimulation with Ang II for 48 h, mRNA expression of ANP and β-MHC increased by 7.79-fold and 2.90-fold, respectively. Treatment with WP1066 significantly blunted the increase in ANP and β-MHC mRNA levels in a dose-dependent manner, indicating that STAT3 inhibition is able to prevent the development of Ang II-induced hypertrophy in H9c2 cells.

### STAT3 inhibition markedly enhanced Ang II-induced myocyte autophagy

**Fig 4:**
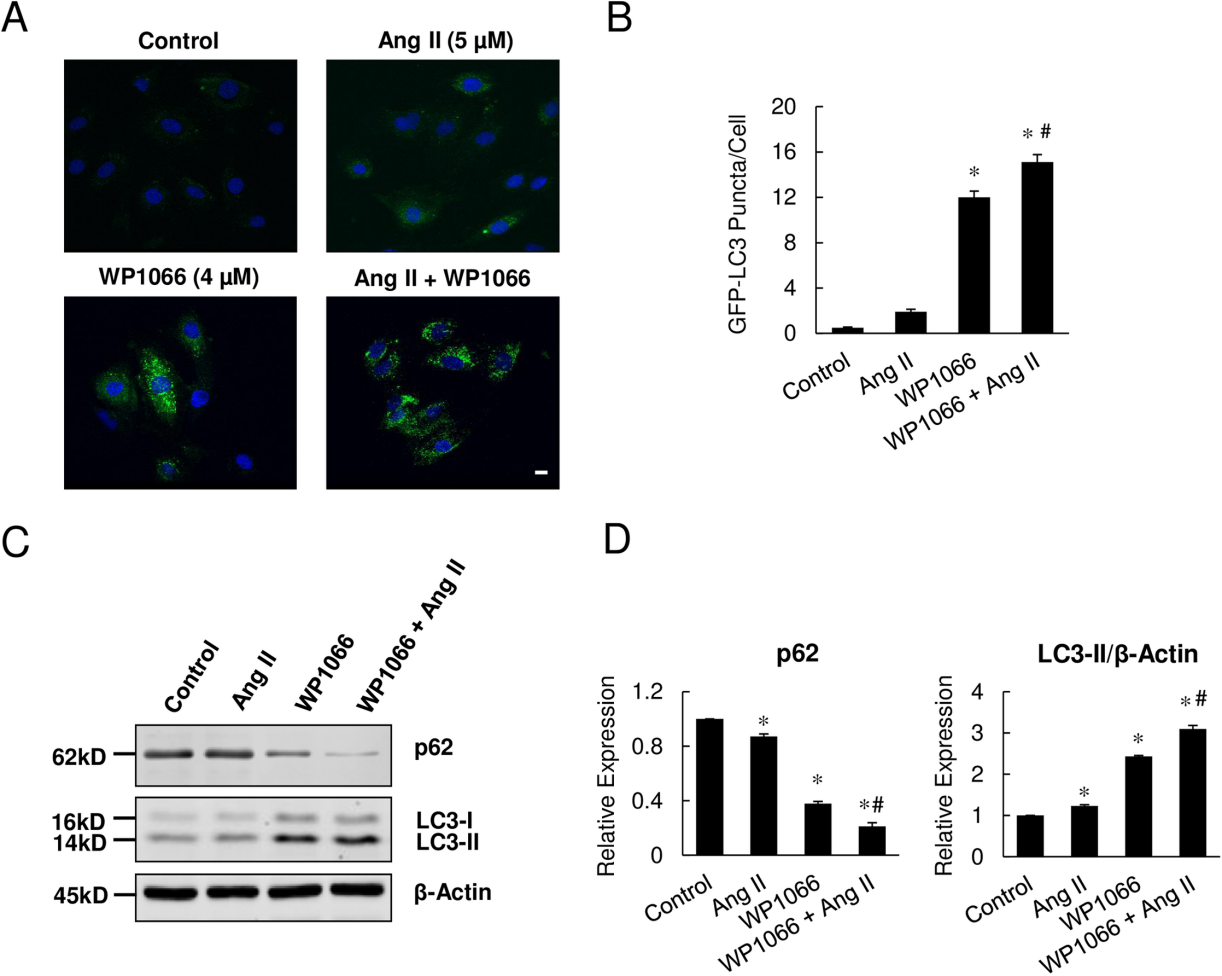
Induction of myocyte autophagy by Ang II and STAT3 inhibition. (A) GFP-LC3 puncta (green) in H9c2 cells transfected with GFP-LC3 and treated with vehicle (Control) or Ang II and WP1066 alone or in combination. Scale bar = 10 μm. (B) Quantitation of GFP-LC3 autophagosomes (green puncta) in H9c2 cells treated with vehicle (Control) or Ang II and WP1066 alone or in combination. (C) Protein expression of autophagy markers p62 and LC3 in H9c2 cells treated with Ang II or/and WP1066. (D) Densitometric analysis of p62 and LC3 protein expression levels. Data represent mean ± SEM (n = 3). *P<0.05 vs. control; ^#^*P*<0.05 vs. Ang II only.

Next we examined whether Ang II induced autophagy in H9c2 cells and whether STAT3 activation played a role in this process. The subcellular localization of EGFP-LC3 protein in H9c2 cells treated with Ang II and/or STAT3 inhibitor WP1066 was analyzed. After stable expression with GFP-LC3, exposure to Ang II alone resulted in a modest increase in EGFP-LC3 puncta (Fig 4A and 4B), a nonsignificant reduction in p62 levels (Fig 4C and 4D), and minimal change in LC3-II levels (Fig 4C and 4D) compared with control cells. However, treatment with WP1066 alone or in combination with Ang II resulted in marked increases in EGFP-LC3 puncta (24-fold and 30.2-fold increase in WP1066 only and Ang II+WP1066 groups, respectively (Fig 4A and 4B). Consistently, the levels of p62 decreased and LC3-II increased significantly in these groups compared with controls (Fig 4C and 4D).

To further elucidate the induction of autophagy, the expression of p62 and LC3-II, molecular markers of autophagy, were examined. The protein levels of p62 decreased significantly in H9c2 cells treated with STAT3 inhibitor WP1066, with further reduction with concomitant exposure to Ang II, indicating marked increase in autophagy (Fig 4C and 4D). Furthermore, treatment with WP1066 alone or in combination with Ang II resulted in marked increases in LC3-II protein levels, indicating robust induction of autophagy in H9c2 cells (Fig 4C and 4D). These findings were further corroborated by electron microscopic observations of increased amounts of autophagolysosomes in STAT3-KD cells compared with scramble control cells (S1A and S1B Fig). Consistently, the expression of LC3-II was noted to be greater in STAT3-KD H9c2 cells, indicating enhancement of autophagy with greatly reduced levels of STAT3 (S1C and S1D Fig). Furthermore, treatment with WP1066 alone increased LC3-II expression in a dose-dependent manner, indicating progressive induction of autophagy with increasing STAT3 inhibition (S2A and S2B Fig). Taken together, these results indicate that exposure to Ang II induces myocyte autophagy, which is markedly enhanced by concomitant inhibition of STAT3.

### STAT3 modulated autophagy through the AMPKα/mTOR signaling pathway

**Fig 5:**
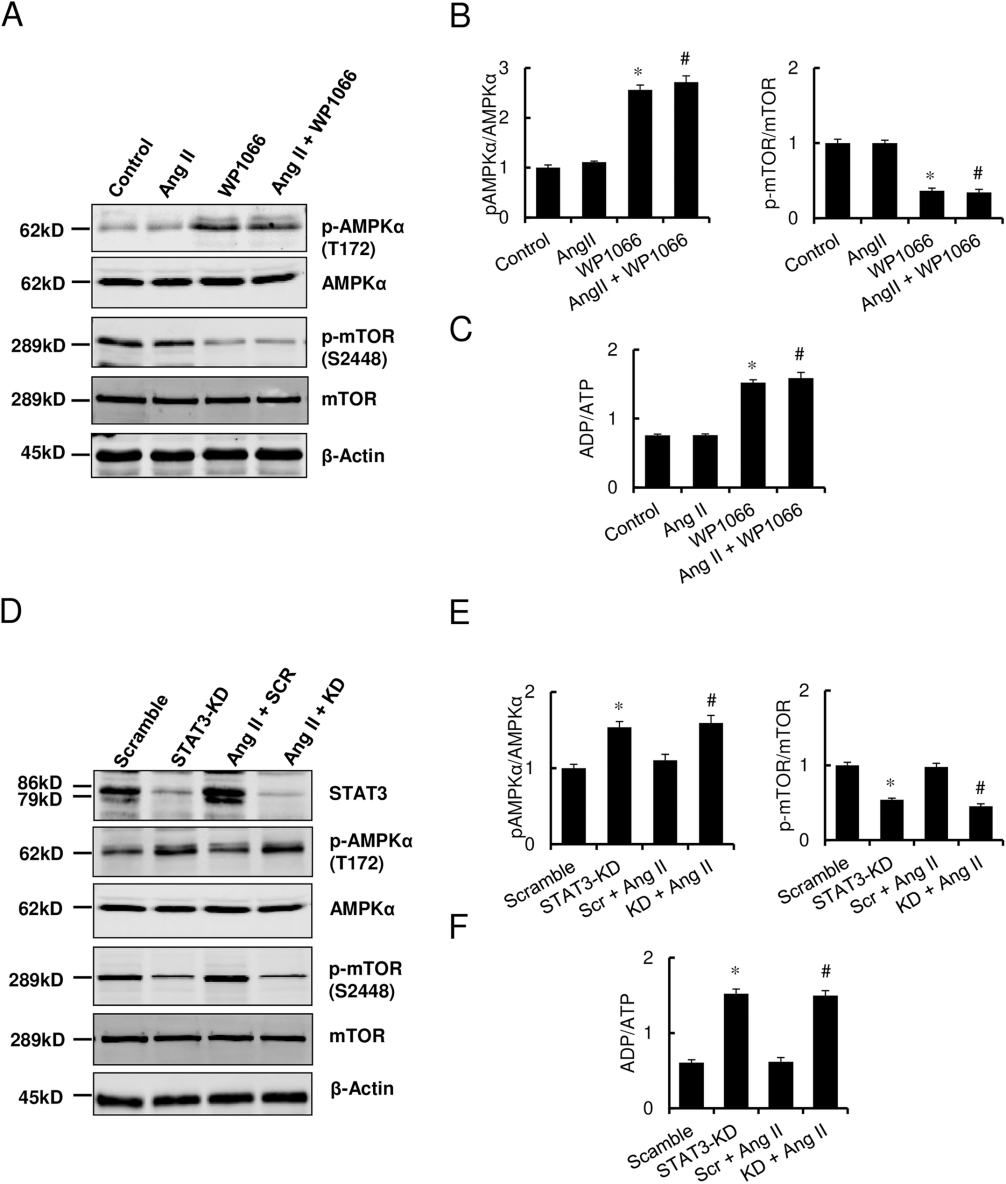
STAT3 modulates autophagy in H9c2 cells via AMPKα/mTOR pathway. (A) Representative Western immunoblots showing p-AMPKα, AMPKα, p-mTOR, mTOR, and β-actin protein expression in H9c2 cells treated with vehicle control, Ang II (5 μM), WP1066 (4μM) alone, and Ang II + WP1066 for 48 h. (B) The densitometric quantitation of p-AMPKα, AMPKα, p-mTOR and mTOR protein levels in H9c2 cells. (C) ADP/ATP ratio in H9c2 cells following indicated treatments. Data represent mean ± SEM (n = 3).*P<0.05 vs. control; ^#^*P*<0.05 vs. Ang II only. (D) Representative Western immunoblots showing STAT3, p-AMPKα, AMPKα, p-mTOR, mTOR, and β-actin protein expression in H9c2 cells treated with scramble peptide control, STAT3 shRNA, scramble RNA + Ang II, and STAT3 shRNA + Ang II for 48 h.(E) The densitometric quantitation of p-AMPKα, AMPKα, p-mTOR and mTOR protein levels in H9c2 cells. (F) ADP/ATP ratio in STAT3-KD cells following Ang II treatment. Data represent mean ± SEM (n = 3).*P<0.05 vs. control (scramble RNA); ^#^*P*<0.05 vs. STAT3-KD only.

To identify the molecular cascade by which STAT3 modulates Ang II-induced hypertrophic response, the AMPKα/mTOR pathway was examined as it has considerable importance in cardiac hypertrophy as well as autophagy. Both pharmacologic (WP1066) and genetic approaches (STAT3-KD cells) were used to inhibit STAT3 in H9c2 cells. As shown in Fig 5A and 5B, treatment with Ang II alone did not alter the levels of p-AMPKα or p-mTOR significantly. Exposure to Ang II alone also failed to affect the ADP/ATP ratio significantly, which further supported the immunoblotting findings with regard to the non-significant impact of Ang II alone on AMPKα and mTOR signaling (Fig 5C).

In contrast, Western immunoblot analysis following STAT3 inhibition with WP1066 revealed significant increase in p-AMPKα levels and significant decrease in p-mTOR levels, consistent with autophagy induction. Densitometric analysis of the immunoreactive bands demonstrated that p-AMPKα levels increased by 1.78-, 3.09- and 4.08-fold with 2 μM, 4 μM, and 8 μM concentrations of WP1066, respectively, compared with control (Fig 5A and 5B, S2A and S2C Fig). In addition, p-mTOR levels decreased significantly to 0.82-, 0.52- and 0.22-fold in cells treated with 2 μM, 4 μM, and 8 μM concentrations of WP1066, respectively, compared with control (Fig 5A and 5B, S2A and S2C Fig). As a key substrate of mTOR kinase, p-4E-BP1 levels also decreased by WP1066 (S2A and S2C Fig), further supporting an essential role of STAT3 in regulation of mTOR function. Further examination of the AMPKα and mTOR activity by Ang II alone and WP1066 co-treatment demonstrated that Ang II did not induce p-AMPKα or suppress p-mTOR expression (Fig 5A and 5B).

The effects of Ang II and STAT3 on AMPKα and mTOR activation were further investigated by the use of the stable STAT3-KD cell line. Knocking down STAT3 significantly activated AMPKα and inhibited mTOR activation (Fig 5D and 5E). To further examine how AMPKα and mTOR activation are affected by Ang II stimulation with or without STAT3, we subjected both scramble and STAT3-KD cells to Ang II. As shown in Fig 5D and 5E, p-AMPKα and p-mTOR levels were not significantly impacted by the addition of Ang II.

In order to elucidate the molecular underpinnings of STAT3-induced AMPKα/mTOR signaling, the ADP/ATP levels were measured. Consistent with our observations with p-AMPKα and p-mTOR levels, STAT3 suppression with WP1066 treatment (Fig 5C) or STAT3 knockdown (Fig 5F) both increased the ADP/ATP ratio significantly. In contrast, exposure to Ang II alone did not induce any significant change in ADP/ATP ratio. When STAT3 suppression was achieved in addition to Ang II treatment, the ADP/ATP ratio was similar to those achieved with STAT3 inhibition alone (Fig 5C and 5F). Taken together, these results indicate that AMPKα and mTOR activities are predominantly regulated by STAT3 during Ang II induced myocyte hypertrophy.

**Fig 6:**
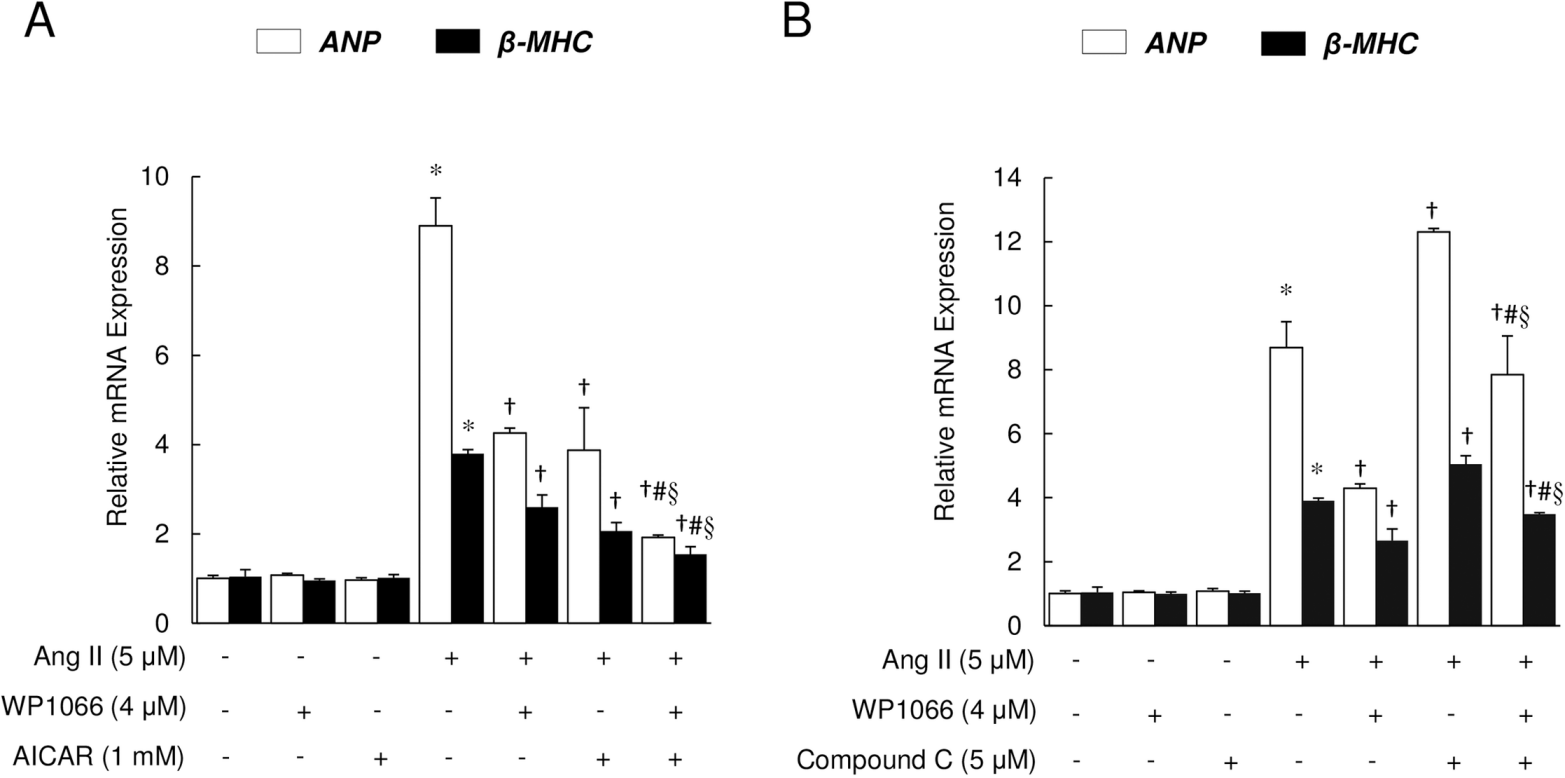
AMPKα signaling is critical for the mediation of STAT3 influence on hypertrophy. (A) WP1066 and AICAR treatment for 48 h suppressed Ang II-induced ANP and β-MHC mRNA expression. Data represent mean ± SEM (n = 4); *P<0.01 vs. Control; ^†^*P*<0.01 vs. Ang II alone; ^#^*P*<0.01 vs. Ang II + WP1066; ^§^*P*<0.05 vs. Ang II + AICAR. (B) Inhibition of AMPK with Compound C reversed the suppression of hypertrophy marker expression by WP1066 following Ang II treatment of H9c2 cells. Data represent mean ± SEM (n = 4); *P<0.01 vs. Control; ^†^*P*<0.01 vs. Ang II alone; ^#^*P*<0.01 vs. Ang II + WP1066; and ^§^*P*<0.05 vs. Ang II + Compound C.

To further elucidate the role of STAT3/AMPKα/mTOR signaling pathway during Ang II-induced myocyte hypertrophy, a specific activator (AICAR) and inhibitor (Compound C) of AMPKα were used in co-treatment experiments with Ang II and WP1066. The mRNA expression of ANP and β-MHC was used as readouts. Exposure to Ang II alone induced a 8.9-fold increase in ANP and 3.8-fold increase in β-MHC mRNA expression levels (Fig 6A and 6B). This Ang II-induced increase in hypertrophy markers was suppressed by co-treatment with AMPKα activator AICAR, with even greater reduction with addition of STAT3 inhibitor WP1066 (Fig 6A). In contrast, inhibition of AMPKα with Compound C accentuated the Ang II-induced increase in hypertrophy marker gene expression, while STAT3 inhibition attenuated these effects of AMPKα inhibition (Fig 6B).

## Discussion

### Salient findings

Although Ang II is well known to induce cardiomyocyte hypertrophy, the details of molecular signaling remains incompletely understood. The primary findings of our studies using Ang II stimulation *in vitro* are as follows: i) Ang II activates STAT3 and induces hypertrophy in H9c2 cells; ii) concomitantly, Ang II also enhances autophagy, however, without any significant impact on AMPKα/mTOR pathway and without altering cellular ADP/ATP ratio; iii) inhibition of STAT3 results in marked increase in autophagy and attenuation of myocyte hypertrophy; and iv) STAT3 inhibition increases myocyte ADP/ATP ratio and activates AMPKα/mTOR pathway. Together, these results identify STAT3 as a key determinant of myocyte hypertrophy vis-à-vis autophagy following Ang II stimulation, through modulation of AMPKα/mTOR signaling.

Signaling initiated by Ang II is complex and leads to numerous physiological and pathological outcomes depending on the context [12]. Although Ang II is known to induce myocyte hypertrophy, the precise signaling events, especially with regard to the role of STAT3 remain unclear [13]. Moreover, the prevailing evidence implicates the transcriptional or genomic actions of STAT3 as the primary drivers of myocyte hypertrophy [14]. In our study, Ang II-induced myocyte hypertrophy was significantly attenuated by pharmacologic or genetic inhibition of STAT3, indicating a key role played by the JAK2-STAT3 pathway in myocyte hypertrophy. STAT3 is an important mediator of cardiac survival pathway [15,16], and participation of STAT3 signaling in various other cardiac physiological or pathological processes, including protection against ischemia [16,17], and hypertrophy [14] has been documented [18,19]. Previous reports have also suggested that JAK2/STAT3 promoted the transition of cardiac hypertrophy to failure [20]. JAK2 is activated by Ang II through Type 1 receptor in a G-protein dependent manner [21]. Phosphorylation and activation of STAT3 is regulated by JAK2 [18,22], consistent with the current findings, which show increased p-JAK2 levels following Ang II exposure. Importantly, Ang II-induced STAT3 phosphorylation was independent of AMPKα and mTOR activity, suggesting that AMPKα/mTOR signaling is limited to the induction of autophagy following STAT3 inhibition, and does not play a significant role in Ang II-induced cardiomyocyte hypertrophy.

Since cellular growth during hypertrophy requires accelerated protein synthesis, the attendant derangements of protein quality control may lead to additional pathologies. Indeed, cardiac hypertrophy may exacerbate ventricular functional decline by producing misfolded and toxic proteins [23]. Consistently, growing evidence indicates that autophagy plays important roles toward myocyte protein homeostasis not only at the basal state, but also during hypertrophy and failure [6]. However, the extents of stimulation or inhibition of cardiac autophagy by Ang II have varied considerably in prior studies [24,25]. The current data show that Ang II treatment increased LC3-II expression, reduced p62 levels, and enhanced (*P* = 0.091) the number of EGFP-LC3 puncta, indicating increased myocyte autophagy concomitant with myocyte hypertrophy. However, and interestingly, Ang II stimulation alone did not affect the activation of AMPKα and mTOR, nor did it alter the ADP/ATP ratio significantly. These observations suggest that Ang II-induced increase in autophagy occurs largely independent of cellular energy status and possibly involves pathways other than the classical AMPKα/mTOR axis.

With regard to the above findings, it is important to consider that Ang II is known to activate a broad array of molecular pathways [12], often in cell-type-specific manners [26]. Indeed, in smooth muscle cells, Ang II stimulation has been shown to increase AMPK activity [27], whereas Ang II infusion reduced AMPK phosphorylation in cardiac fibroblasts *in vivo* [28]. In H9c2 cells, Ang II stimulation produced minimal and nonsignificant elevation in p-AMPKα/AMPK ratio [29], similar to the current observations. Although the precise reasons for these cell-type specific differences in Ang II effects on AMPK activation remain to be deciphered, the current findings serve to further underscore the complexity of Ang II signaling in different cells and tissues.

Another key finding from this investigation demonstrates that inhibition of STAT3 is associated with induction of autophagy in Ang II-treated H9c2 cells. Pharmacologic blockade or genetic silencing of STAT3 in effect converted the myocyte response to Ang II from predominantly hypertrophic to largely autophagic. These observations indicate that STAT3 signaling is a critical determinant of the overall structural outcomes of Ang II stimulation in myocytes. These results are also consistent with the prior evidence that induction of autophagy prevents the development of cardiac hypertrophy or regresses established hypertrophy [5,7]. Enhanced autophagy through STAT3 inhibition may therefore prove to be an important therapeutic modality in patients with cardiac hypertrophy.

Furthermore, our results demonstrate that inhibition of STAT3 results in enhanced autophagy through activation of AMPKα and consequent inhibition of mTOR signaling. mTOR is known to be an important regulator of protein synthesis, and mTOR inhibition is associated with induction of autophagy [9]. The current data show that suppression of STAT3 induces autophagy by inhibiting mTOR activity in H9c2 cells, suggesting that STAT3 modulates autophagy through an mTOR-dependent mechanism. AMPKα, which is upstream of mTOR [30], served as the molecular link between STAT3 inhibition and mTOR inhibition. AMPKα acts as a sensor of cellular metabolic status and regulates autophagy under diverse physiological and pathological scenarios [30]. Previous studies have found that activation of AMPKα inhibits cardiac hypertrophy [31]. The current observations with both pharmacological and genetic inhibition of STAT3 unravel a heretofore unknown role of STAT3 signaling in regulation of AMPKα/mTOR. While mTOR has been shown to regulate STAT3/p63/Notch signaling in cancer cells [32], the molecular details of STAT3 modulation of AMPKα and/or mTOR activity remain unknown. However, *in vivo* studies of hypothalamus with regard to food intake have suggested the possibility that AMPK might function downstream of or in a pathway parallel to STAT3 [33]. Importantly, the current observations were further substantiated by findings from studies of AMPKα activation and inhibition by AICAR and Compound C, respectively. The decrease in Ang II-induced mRNA expression of hypertrophy markers ANP and β-MHC with AMPKα activator AICAR was further accentuated by STAT3 inhibitor WP1066. Congruently, the augmentation of hypertrophy gene expression by the AMPKα inhibitor Compound C was blunted by co-treatment with WP1066. Taken together, our data suggest that a novel putative STAT3/AMPKα/mTOR signaling pathway balances Ang II-induced myocyte hypertrophy and autophagy.

Both pharmacologic and genetic inhibition of STAT3 resulted in significant increase in cellular ADP/ATP ratio, indicating myocyte ATP depletion. Since AMPKα is activated by reduction in cellular energy source, this alteration in cellular ATP levels may represent the underlying mechanism for the activation of AMPKα with STAT3 inhibition. In the myocardium, AMPKα activation boosts ATP production by stimulating both fatty acid oxidation and glycolysis, while slowing down ATP-consumption processes [30,34]. In addition, AMPKα also influences myocyte energetics by activating PGC-1α and increasing mitochondrial biogenesis and oxidative capacity [30,35]. Thus, activation of AMPKα by STAT3 suppression may also improve mitochondrial and myocardial energetics, protein homeostasis, and attenuation of cardiomyocyte hypertrophy through increased autophagy.

STAT3 participates in numerous signaling pathways in the cardiovascular system culminating in transcriptional responses that are largely protective, reparative, and prosurvival. These genomic actions of STAT3 in the heart have been shown to upregulate antiapoptotic, antioxidant and proangiogenic genes [18,19,22]. In contrast, persistent and excessive STAT3 activation has been linked to adverse outcomes, including increased inflammation, ventricular rupture, and heart failure [36]. Similarly, prolonged STAT3 activation during pathogenesis of cardiac hypertrophy may prove harmful for long-term preservation of cardiac function due to eventual functional deterioration of hypertrophic myocytes. Interestingly, our results suggest that STAT3 inhibition rescues Ang II-induced myocyte hypertrophy through effects on cellular energy status, which are regulated by nongenomic actions of STAT3. Although the signaling details remain incompletely understood, growing evidence indicates that mitochondrial STAT3 mediates critical functions in cellular respiration and survival [10,19]. The current data therefore suggest that transcriptional and nongenomic effects of STAT3 are interdependent with regard to pathogenesis of cardiomyocyte hypertrophy.

In summary, our results unravel complex signaling details with regard to the role of STAT3 in Ang II-induced myocyte hypertrophy. While Ang II stimulation led to STAT3 activation, myocyte hypertrophy, and enhanced autophagy, inhibition of STAT3 triggered autophagy and attenuated hypertrophy through increased ADP/ATP ratio and activation of AMPKα. These results suggest interdependence of genomic and nongenomic actions of STAT3 with regard to myocyte protein and energy homeostasis and myocyte growth. Thus, modulation of STAT3/AMPKα/mTOR axis may prove useful for treatment of RAS-mediated cardiovascular pathologies.

## Supporting information

S1 Fig Enhanced induction of autophagy in H9c2 cells after STAT3 knockdown (STAT3-KD). Representative electron microscopic images of autophagolysosomes (arrows) in H9c2 cells treated with scramble peptide (A) and STAT3 shRNA (STAT3-KD, B). (C) Western immunoblots showing LC3-I and -II expression in H9c2 cells treated with scramble peptide or STAT3 shRNA. (D) Densitometric quantitation of LC3-I and -II levels. Data represent mean ± SEM (n = 3). *P< 0.05 vs. Scramble control.
(TIF)

S2 Fig Dose dependent activation of AMPKα/mTOR signaling pathway by STAT3 suppression. (A) Representative Western immunoblots showing p-AMPKα, AMPKα, p-mTOR, mTOR, p-4E-BP1, LC3-I, LC3-II, and β-actin protein expression in H9c2 cells treated with increasing concentration of WP1066 for 48 h. Densitometric quantitation of protein levels of autophagy marker LC3-II (B) and p-AMPKα and p-mTOR (C). Data represent mean ± SEM (n = 3). *P<0.05 vs. control.
(TIF)
