# Correction: STAT3 balances myocyte hypertrophy vis-à-vis autophagy in response to Angiotensin II by modulating the AMPKα/mTOR axis

**DOI:** 10.1371/journal.pone.0294366

**Published:** 2023-11-08

**Authors:** Lei Chen, Lin Zhao, Anweshan Samanta, Seyed Morteza Mahmoudi, Tanner Buehler, Amy Cantilena, Robert J. Vincent, Magdy Girgis, Joshua Breeden, Samuel Asante, Yu-Ting Xuan, Buddhadeb Dawn

The AMPKα panel in Fig 5A [1] is incorrect as it presents an inadvertent duplication of the AMPKα panel in Fig 5D. The AMPKα panel in Fig 5A is replaced in the updated Fig 5 provided with this notice. Original western blot images and quantitative data underlying the results in this figure are provided in S1 and S2 Files.

**Fig 5:**
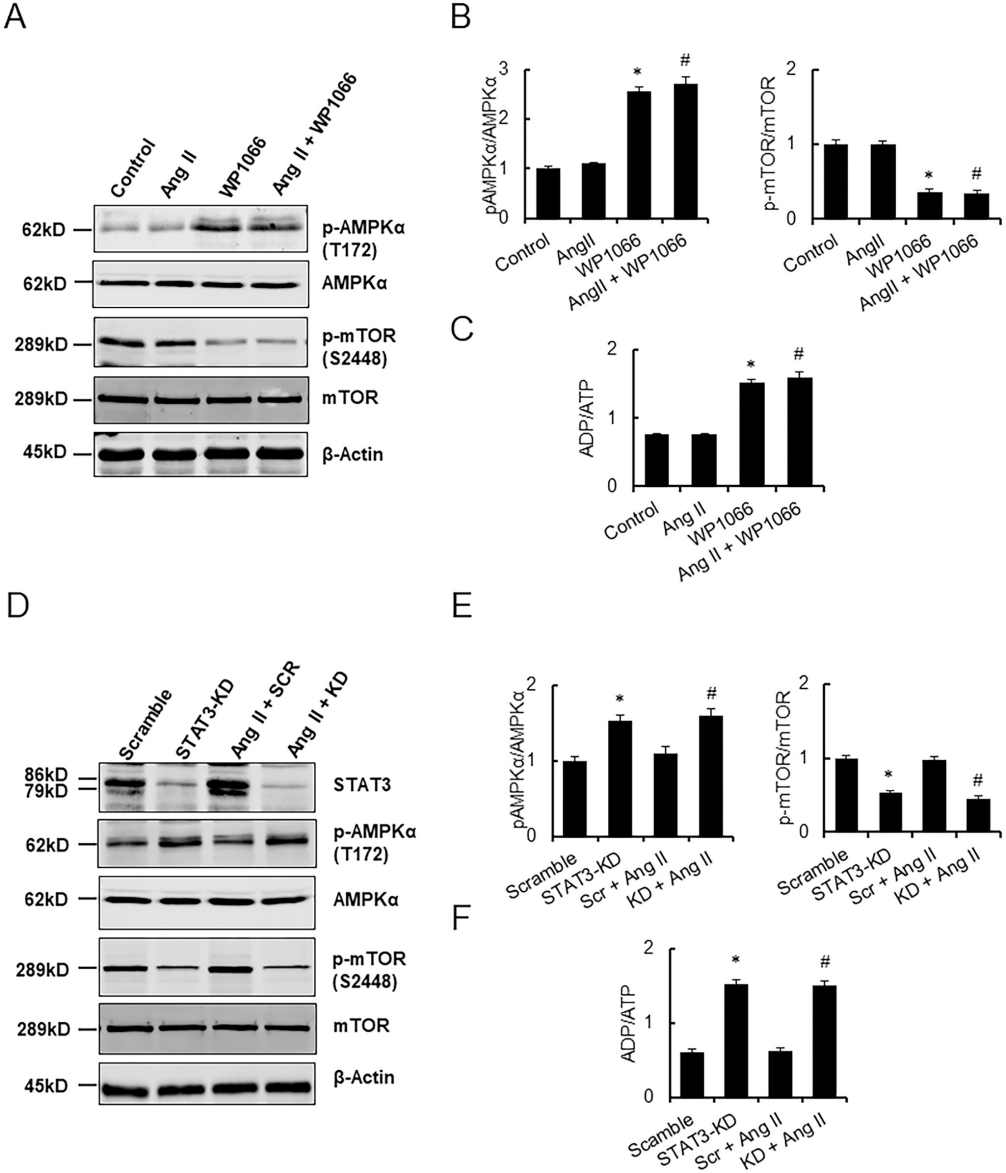
STAT3 modulates autophagy in H9c2 cells via AMPKα/mTOR pathway. (A) Representative Western immunoblots showing p-AMPKα, AMPKα, p-mTOR, mTOR, and β-actin protein expression in H9c2 cells treated with vehicle control, Ang II (5 μM), WP1066 (4μM) alone, and Ang II + WP1066 for 48 h. (B) The densitometric quantitation of p-AMPKα, AMPKα, p-mTOR and mTOR protein levels in H9c2 cells. (C) ADP/ATP ratio in H9c2 cells following indicated treatments. Data represent mean ± SEM (n = 3). *P<0.05 vs. control; *#P*<0.05 vs. Ang II only. (D) Representative Western immunoblots showing STAT3, p-AMPKα, AMPKα, p-mTOR, mTOR, and β-actin protein expression in H9c2 cells treated with scramble peptide control, STAT3 shRNA, scramble RNA + Ang II, and STAT3 shRNA + Ang II for 48 h.(E) The densitometric quantitation of p-AMPKα, AMPKα, p-mTOR and mTOR protein levels in H9c2 cells. (F) ADP/ATP ratio in STAT3-KD cells following Ang II treatment. Data represent mean ± SEM (n = 3). *P<0.05 vs. control (scramble RNA); *#P*<0.05 vs. Scr + AngII.

An additional error in the figure legend is also corrected: “^*#*^*P*<0.05 vs. STAT3-KD only” is changed to “^*#*^*P*<0.05 vs. Scr + AngII.”

In addition, the primary data underlying results in this article were not included with the published article although the Data Availability statement for this article stated, “All relevant data are within the paper and its Supporting Information files.” With this Correction, the authors clarify that the original raw data are available on request from the corresponding author.

The authors apologize for the error in the published article.

## Supporting information

S1 File Original western blot images for Fig 5A and 5D. (PPTX)

S2 File Quantitative data underlying Fig 5B, 5C, 5E and 5F. (XLSX)
