# Supplementary figures and images for: STAT3 balances myocyte hypertrophy vis-à-vis autophagy in response to Angiotensin II by modulating the AMPKα/mTOR axis
Source: PLoS One. 2017 Jul 7;12(7):e0179835. doi: 10.1371/journal.pone.0179835 (PMC5501431; doi:10.1371/journal.pone.0179835)

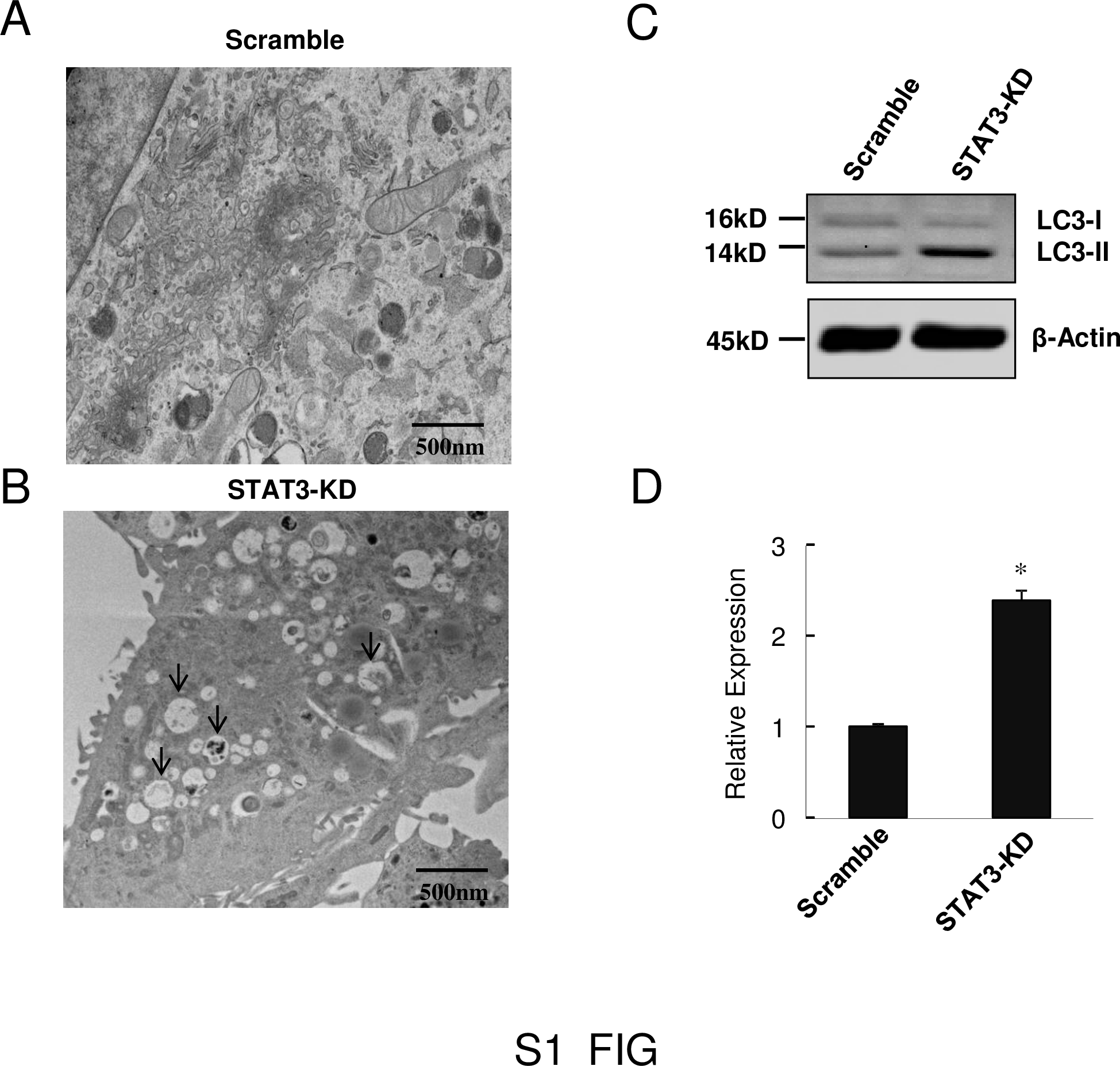

Supplement: S1 Fig — Representative electron microscopic images of autophagolysosomes (arrows) in H9c2 cells treated with scramble peptide (A) and STAT3 shRNA (STAT3-KD, B). (C) Western immunoblots showing LC3-I and -II expression in H9c2 cells treated with scramble peptide or STAT3 shRNA. (D) Densitometric quantitation of LC3-I and -II levels. Data represent mean ± SEM (n = 3). *P< 0.05 vs. Scramble control. (TIF) [file pone.0179835.s001.tif]

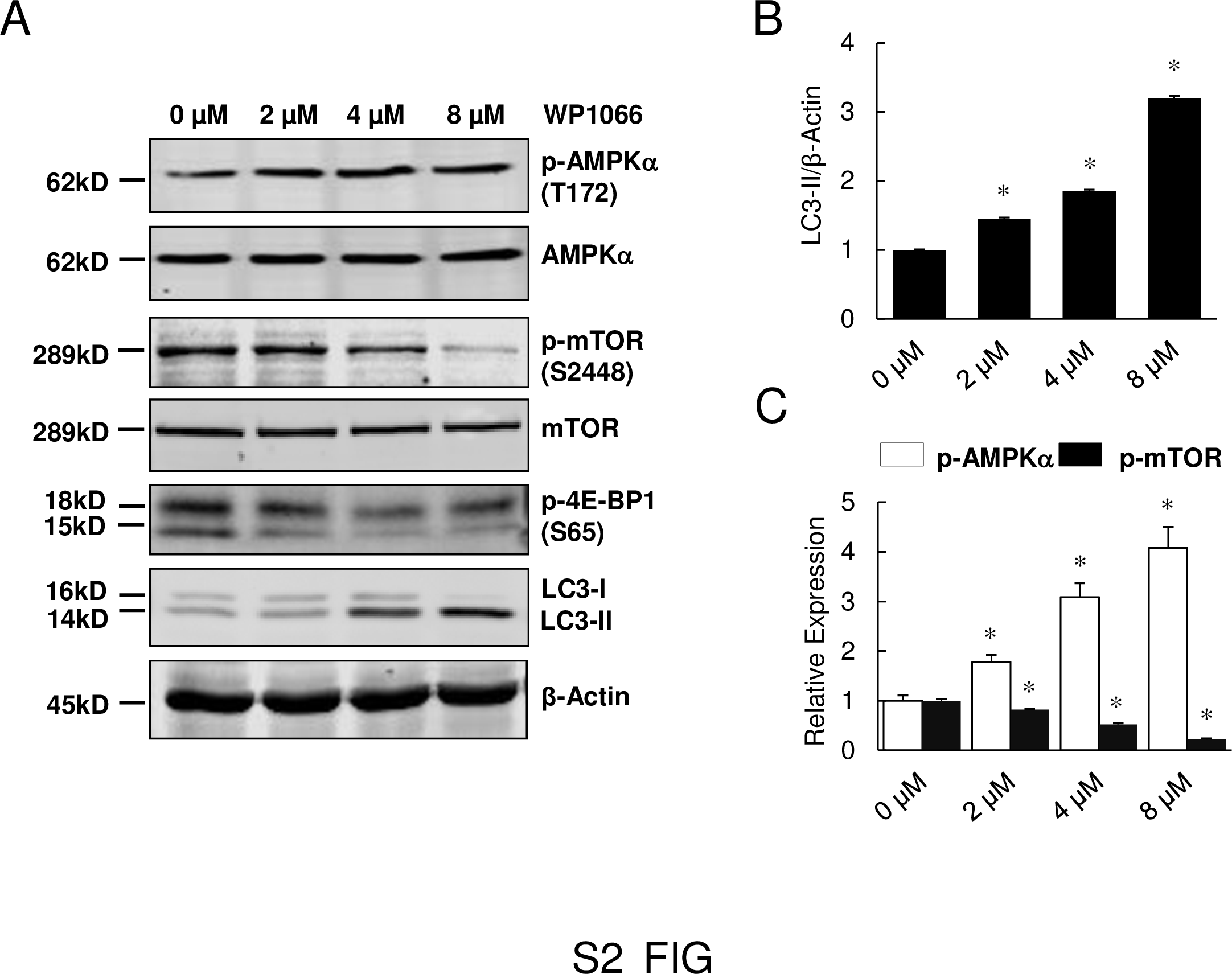

Supplement: S2 Fig — (A) Representative Western immunoblots showing p-AMPKα, AMPKα, p-mTOR, mTOR, p-4E-BP1, LC3-I, LC3-II, and β-actin protein expression in H9c2 cells treated with increasing concentration of WP1066 for 48 h. Densitometric quantitation of protein levels of autophagy marker LC3-II (B) and p-AMPKα and p-mTOR (C). Data represent mean ± SEM (n = 3). *P<0.05 vs. control. (TIF) [file pone.0179835.s002.tif]
