# Supplementary material for: Correction: STAT3 balances myocyte hypertrophy vis-à-vis autophagy in response to Angiotensin II by modulating the AMPKα/mTOR axis
Source: PLoS One. 2023 Nov 8;18(11):e0294366. doi: 10.1371/journal.pone.0294366 (PMC10631643; doi:10.1371/journal.pone.0294366)
Supplement: S1 File — (PPTX) [file pone.0294366.s001.pptx]

## Slide 1
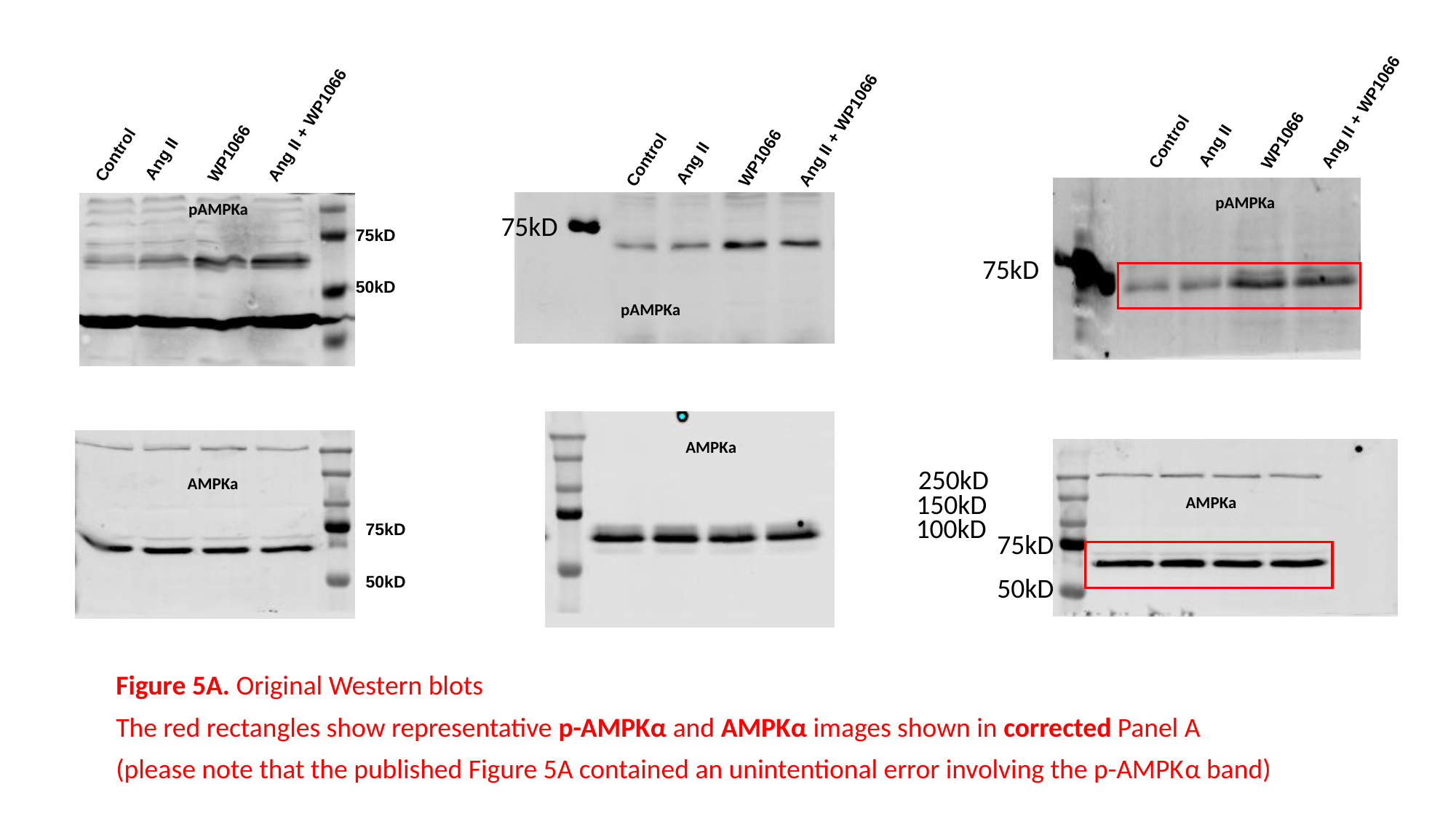

Ang II + WP1066
Ang II
WP1066
Control
pAMPKa
75kD
250kD
150kD
AMPKa
100kD
75kD
50kD
Ang II + WP1066
Ang II
WP1066
Control
pAMPKa
75kD
50kD
AMPKa
75kD
50kD
Ang II + WP1066
Ang II
WP1066
Control
75kD
pAMPKa
AMPKa
Figure 5A. Original Western blots
The red rectangles show representative p-AMPKα and AMPKα images shown in corrected Panel A
(please note that the published Figure 5A contained an unintentional error involving the p-AMPKα band)

## Slide 2
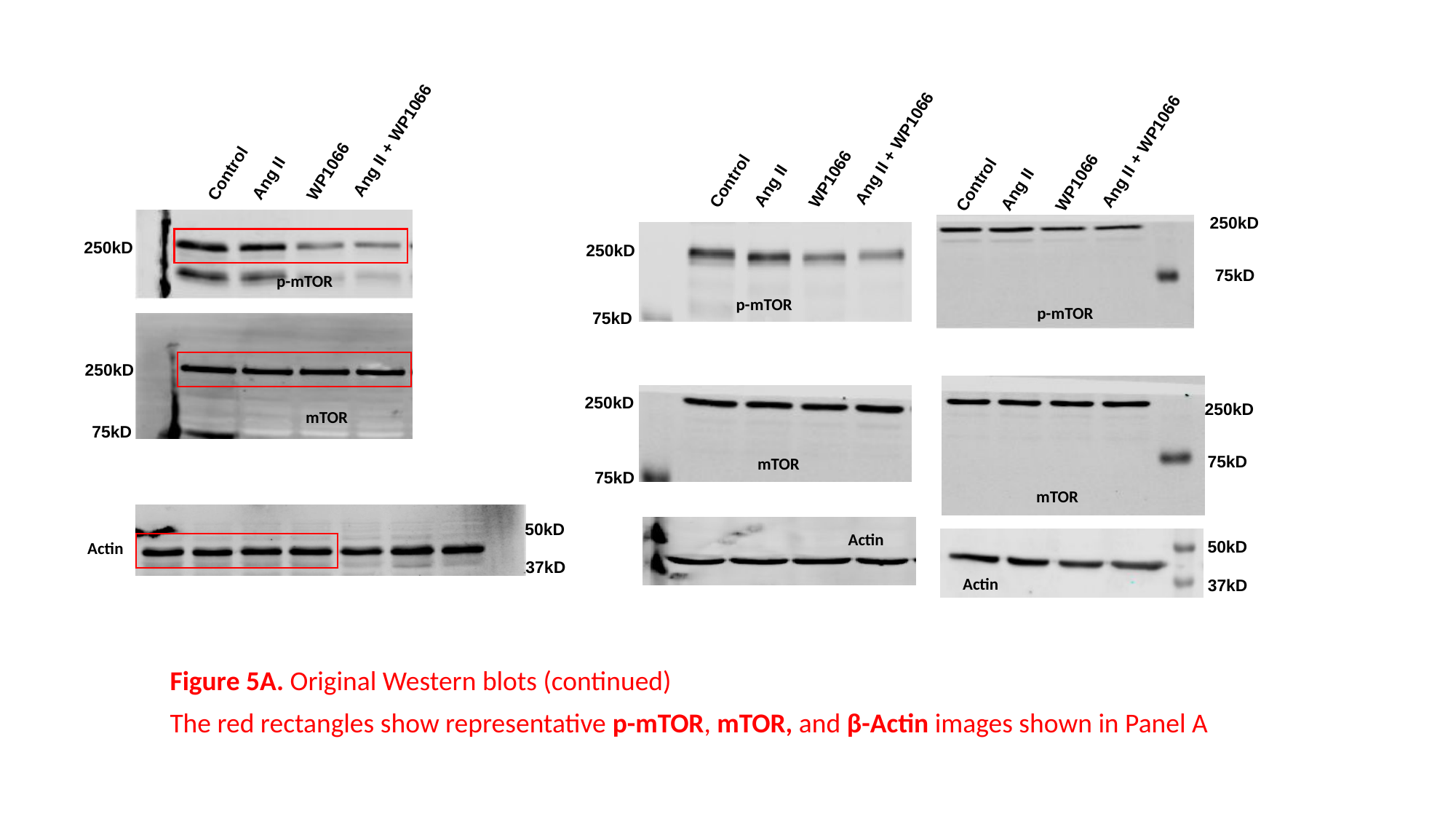

Ang II + WP1066
Ang II + WP1066
Ang II
WP1066
Ang II + WP1066
Control
Ang II
WP1066
Ang II
WP1066
Control
Control
250kD
250kD
250kD
75kD
p-mTOR
p-mTOR
p-mTOR
75kD
250kD
250kD
250kD
mTOR
75kD
75kD
mTOR
75kD
mTOR
50kD
Actin
50kD
Actin
37kD
Actin
37kD
Figure 5A. Original Western blots (continued)
The red rectangles show representative p-mTOR, mTOR, and β-Actin images shown in Panel A

## Slide 3
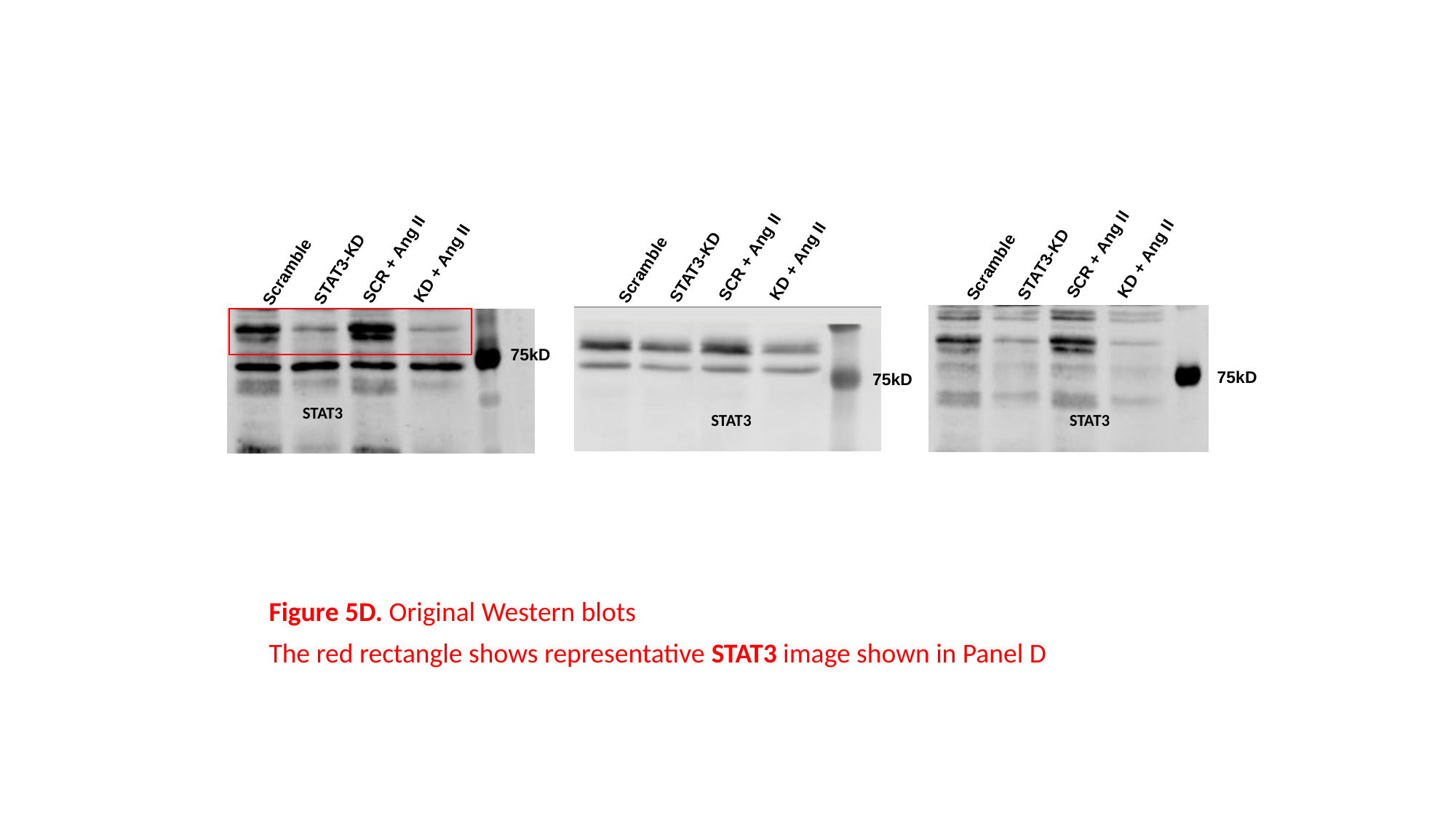

KD + Ang II
STAT3-KD
KD + Ang II
SCR + Ang II
KD + Ang II
STAT3-KD
Scramble
SCR + Ang II
STAT3-KD
SCR + Ang II
Scramble
Scramble
75kD
75kD
75kD
STAT3
STAT3
STAT3
Figure 5D. Original Western blots
The red rectangle shows representative STAT3 image shown in Panel D

## Slide 4
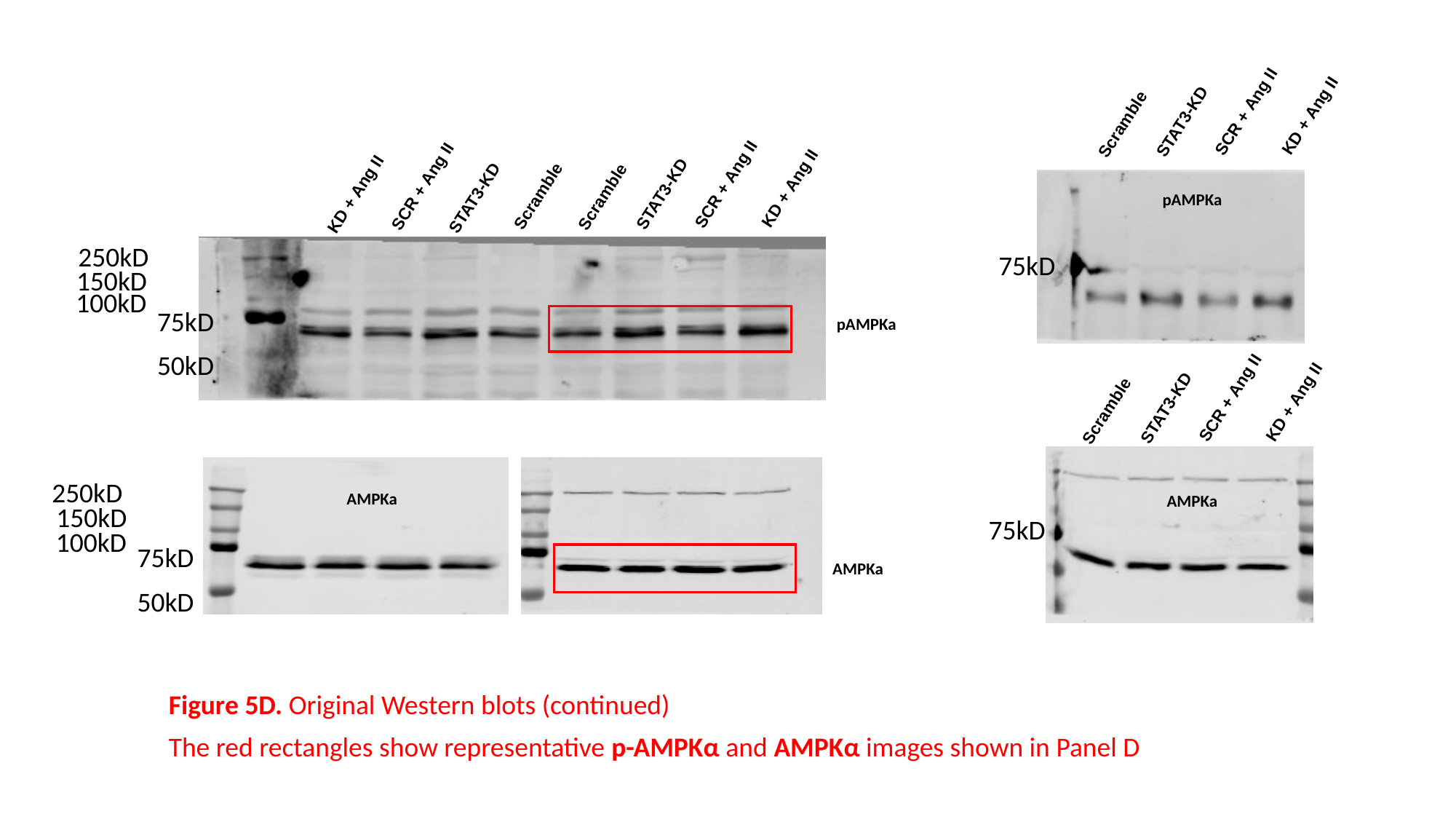

KD + Ang II
STAT3-KD
SCR + Ang II
Scramble
KD + Ang II
STAT3-KD
SCR + Ang II
Scramble
KD + Ang II
STAT3-KD
SCR + Ang II
Scramble
pAMPKa
250kD
75kD
150kD
100kD
75kD
pAMPKa
50kD
KD + Ang II
STAT3-KD
SCR + Ang II
Scramble
250kD
AMPKa
AMPKa
150kD
75kD
100kD
75kD
AMPKa
50kD
Figure 5D. Original Western blots (continued)
The red rectangles show representative p-AMPKα and AMPKα images shown in Panel D

## Slide 5
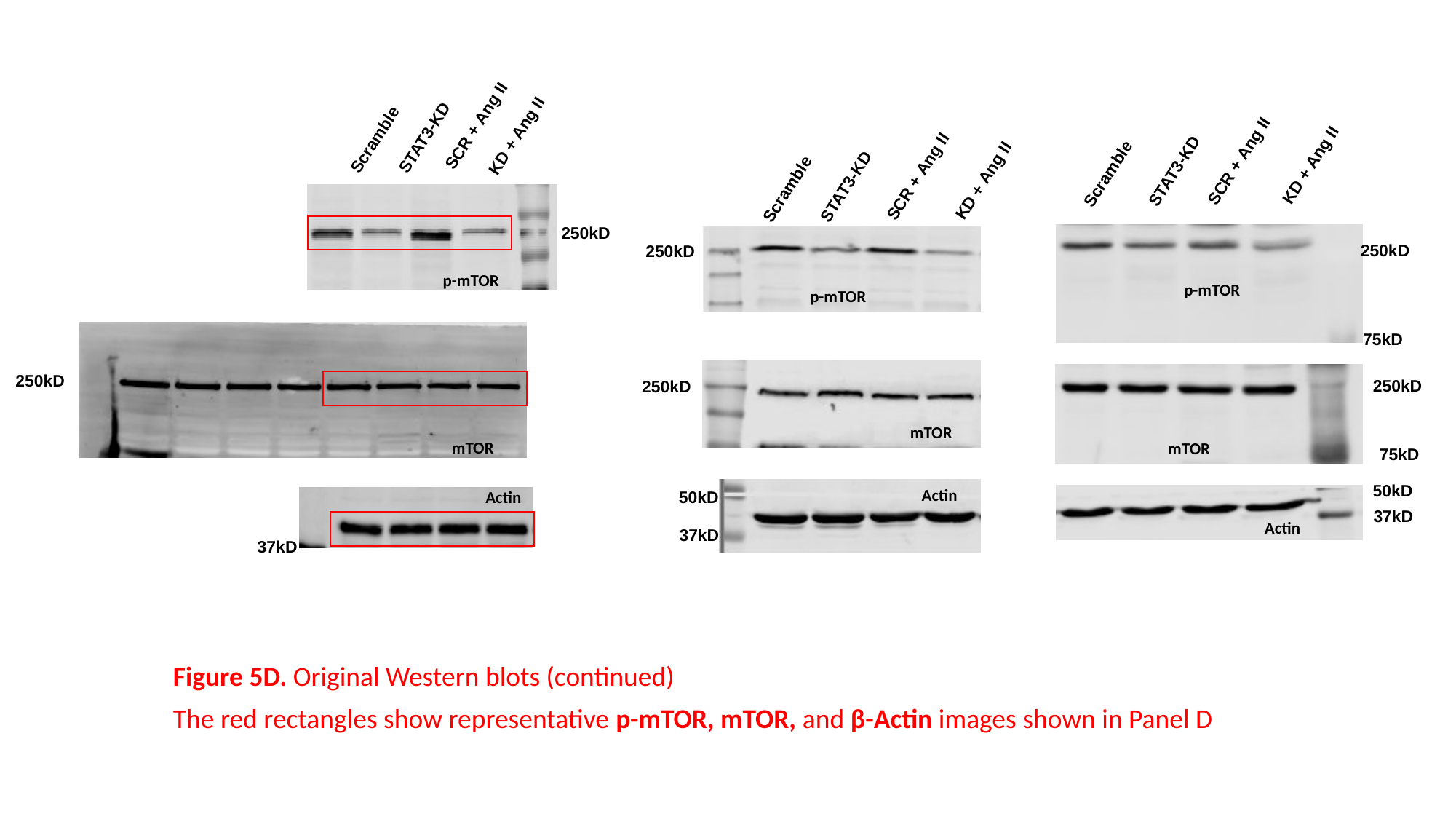

STAT3-KD
SCR + Ang II
KD + Ang II
Scramble
KD + Ang II
STAT3-KD
SCR + Ang II
Scramble
KD + Ang II
STAT3-KD
SCR + Ang II
Scramble
250kD
250kD
250kD
p-mTOR
p-mTOR
p-mTOR
75kD
250kD
250kD
250kD
mTOR
mTOR
mTOR
mTOR
75kD
50kD
Actin
Actin
50kD
37kD
Actin
37kD
37kD
Figure 5D. Original Western blots (continued)
The red rectangles show representative p-mTOR, mTOR, and β-Actin images shown in Panel D
